# Puberty interacts with sleep and brain network organization to predict mental health

**DOI:** 10.3389/fnhum.2024.1379945

**Published:** 2024-09-27

**Authors:** Mackenzie E. Mitchell, Tehila Nugiel

**Affiliations:** ^1^Department of Psychology and Neuroscience, University of North Carolina at Chapel Hill, Chapel Hill, NC, United States; ^2^Department of Psychology, Florida State University, Tallahassee, FL, United States

**Keywords:** puberty, sleep, brain network organization, internalizing, externalizing

## Abstract

**Introduction:**

Along with pubertal development, the transition to adolescence brings about increased risk for sleep disturbances and mental health problems. Functional connectivity of overlapping large-scale brain networks, such as increased connectivity between the default mode and dorsal attention networks, has been reported to relate to both sleep and mental health problems. Clarifying whether pubertal development interacts with sleep disturbances and functional brain networks to predict mental health may provide information to improve the timing and design of interventions targeting sleep disturbances in adolescents.

**Methods:**

To examine how pubertal status and tempo relate to sleep disturbances and shape the relationship between sleep disturbances and mental health problems, we harnessed a large sample of children aged 10–14 years from the Adolescent Brain and Cognitive Development (ABCD) Study (*N* ~ 3,000–10,000). We used graph theoretical tools to probe how pubertal development concurrently interacts with sleep disturbances and brain network organization to predict mental health problems.

**Results:**

We found that advanced pubertal status, but not pubertal tempo, predicted sleep disturbances; however, both pubertal status and tempo interact with sleep disturbances to predict mental health problems and engage in three-way interactions with sleep and brain network organization to predict mental health problems.

**Discussion:**

Overall, this work suggests that less advanced pubertal status and slower tempo are risk factors for the strongest links between sleep disturbances, brain organization, and mental health problems. Further, our findings speak to the importance of accounting for interactions in the constellation of factors that surround complex behavioral and clinical syndromes, here internalizing and externalizing disorders, and provide new context to consider for targeted interventions.

## Introduction

Adolescence is a time of significant change, marked with both opportunities and challenges. Puberty, which serves as a marker denoting the end of childhood and beginning of adolescence, brings about a cascade of physical, social, and behavioral changes (Pfeifer and Allen, 2021). Neurocognitive models of adolescence describe a period of imbalance (Casey, 2015) wherein limbic brain systems that support emotion and reward processing mature more rapidly than prefrontal brain systems that support effortful control (Casey et al., 2008; Blakemore et al., 2010; Gracia-Tabuenca et al., 2021). This imbalance poses adolescence as a particularly vulnerable period for youth. Mental health problems, such as depression and anxiety, exhibit a marked increase during adolescence (Paus et al., 2008; Costello et al., 2011; Mendle, 2014; Powers and Casey, 2015; Solmi et al., 2022). At the individual level, these changes in mental health have been linked to function and change of large scale brain systems, such as the aforementioned systems that support effortful control (Ernst et al., 2019; Goddings et al., 2019; Kircanski et al., 2019). Understanding how these maturational shifts in brain systems can give rise to mental health problems is crucial for better intervention and support for adolescents. There is a constellation of behaviors that evolve during adolescence (Mendle, 2014) that may interact with and even explain the relationship between brain function and mental health. One candidate behavior is sleep. For example, poor quality or insufficient sleep may impact brain function and thus exacerbate mental health problems (Telzer et al., 2013; Yang et al., 2022).

During adolescence, changes in pubertal hormones are tightly linked with a shift in circadian rhythms (Carskadon et al., 1993; Lucien et al., 2021). Adolescents require more sleep for ideal functioning than children and adults (Wolfson and Carskadon, 1998). Yet, at the same time, social, academic and work demands place new constraints on adolescent sleep time (Dahl, 1999). For example, youth who spend more time socializing with friends, studying, or watching TV sleep less on school nights (Fuligni and Hardway, 2006). Adolescents also engage in behaviors that do not promote sleep, such as heavy social media use at night (Alonzo et al., 2021). Pairing these biological and psychosocial pressures to sleep later with the need to wake up early for school creates what has been formalized as the ‘perfect storm’ of adolescent sleep (Carskadon, 2011; Crowley et al., 2018). This need for more sleep paired with the constraints on sleep time are another form of imbalance during adolescence. Concordantly, during adolescence poor sleep quality is related to worse academic and mental health outcomes (Owens et al., 2014; Hysing et al., 2016; Medic et al., 2017; Goldstone et al., 2020). There are neurobiological links between sleep and mental health problems, as well. Connectivity of functional brain networks, including those involved in attention and self-referential processing (i.e., default mode), are related to both mental health burden and sleep in adolescents (Telzer et al., 2013; Yang et al., 2022, 2023). Further, brain network connectivity mediates the relationship between sleep and mental health, such that less segregation of attention and default mode networks exacerbates the relationship between sleep disturbances and current mental health problems, as well as mental health problems a year later (Yang et al., 2022). This work creates a link between sleep, brain function, and mental health in adolescence.

Sleep habits are malleable, and several interventions focused on improving sleep have found moderate improvements in youth sleep quality (Blake et al., 2016, 2017; Becker et al., 2022) and, critically, improvements in mental health (Blake and Allen, 2020; Bourchtein et al., 2020; Scott et al., 2021). Given that changes to sleep habits and mental health both unfold across pubertal development (Lucien et al., 2021; Solmi et al., 2022), we posit that pubertal development may play a moderating role on the relationship between sleep and mental health. If that is the case, the ideal timing of sleep interventions aimed at improving mental health for adolescents may depend on the stage and pace, or ‘tempo’, of puberty. For example, one possibility is that in later stages of puberty, the relationship between sleep and mental health may be stronger. It is also possible that faster pubertal tempo may induce faster shifts in sleep habits, bigger changes in brain function, and higher mental health burden. Understanding how pubertal stage and tempo moderate the links between sleep, brain function, and mental health can inform the appropriate timing and approaches for intervening on sleep habits.

The current study leverages a large cohort of adolescents at various stages of puberty from the Adolescent Brain and Cognitive Development (ABCD) study to examine how pubertal stage and tempo moderate the relationship between sleep quality, mental health problems, and functional brain network connectivity. We focused on integration and segregation of networks that are linked to psychopathology (Peters et al., 2016; Yang et al., 2022; Zhu and Qiu, 2022) and poor sleep (Telzer et al., 2013; Yang et al., 2022) in adolescence. We hypothesize that: (1) Earlier pubertal stages and slower pubertal tempo will be related to better sleep quality; (2) Pubertal stage and tempo will moderate the relationship between sleep and mental health problems, such that the relationship between sleep quality and mental health problems will be weaker for individuals in earlier stages of puberty and for those who have slower puberty tempo; and (3) We will see a three-way interaction between sleep, pubertal measures, and functional brain network connectivity predicting mental health problems. Together, this work stands to clarify how pubertal stage and timing interplay with sleep and its effects on brain function and mental health. For example, it is possible that sensitivity to the negative impacts of sleep disturbances on mental health problems differs across pubertal development. Such clarifications can inform the timing of sleep interventions to promote better mental health outcomes for youth in a vulnerable period.

## Methods

### Participants

In the current study, we used behavioral and neuroimaging data from the ABCD study (Barch et al., 2018; Casey et al., 2018) Data Release 5.0 (doi: 10.15154/8873-ZJ65). The ABCD study is a longitudinal, multi-site, demographically diverse group of ~11,000 adolescents. We used neuroimaging, mental health, and sleep disturbance data at the second follow-up (‘follow-up year two’) when participants were in early stages of puberty (ages 10–14 years) and mental health and sleep problems are known to increase (Costello et al., 2011; Owens et al., 2014).

Neuroimaging data used for this study came from the ABCD-BIDS Collection 3165.^1^ Participants were included in the study if they passed quality assurance checks (see Image acquisition and processing) and had at least 5 min of resting state data after processing. Participants were also excluded if they did not have at least three timepoints of each behavioral measure (see Measures). As such, our final sample for the behavioral models was *N* = 10,596, while our final sample for the models including brain data was *N* = 2,901. Sample demographics can be seen in Table 1.

**Table 1:** Demographics and behavioral measures from the follow-up year 2 collection.

	Total sample for behavioral models (*N* = 10,596)	Sample for models including brain data (*N* = 2,901)
Age (months)	Mean = 144.31, SD = 8.01, range = 127–168	Mean = 143.88, SD = 7.67, range = 127–164
Age (years)	Mean = 12.02, SD = 0.67, range = 10.58–14.00	Mean = 11.99, SD = 0.64, range = 10.58–13.67
Sex		
Female	5,023 (47.4%)	1,460 (50.3%)
Male	5,573 (52.6%)	1,441 (49.7%)
Race/ethnicity		
White	5,756 (54.3%)	1,832 (63.2%)
Black	1,452 (13.7%)	242 (8.3%)
Hispanic	2,059 (19.4%)	534 (18.4%)
Asian	218 (2.1%)	56 (1.9%)
Other	1,111 (10.5%)	237 (8.2%)
Parent marital status		
Married	7,209 (68.0%)	2,168 (74.7%)
Widowed	110 (1.0%)	19 (0.7%)
Divorced	1,061 (10.0%)	265 (9.1%)
Separated	395 (3.7%)	93 (3.2%)
Never married	1,132 (10.7%)	201 (6.9%)
Living with partner	689 (6.5%)	155 (5.3%)
Socioeconomic status (composite score)	Mean = 0.02, SD = 0.88, range = −3.06–1.45	Mean = 0.20, SD = 0.76, range = −2.71–1.45
Sleep disturbances score	Mean = 36.29, SD = 8.00, range = 26–105	Mean = 35.96, SD = 7.59, range = 26–84
PDS score (‘status’)^*^	Mean = 2.17, SD = 0.72, range = 1–4	Mean = 2.13, SD = 0.70, range = 1–4
PDS slope (‘tempo’)	Mean = 0.48, SD = 0.14, range = −0.14–0.92	Mean = 0.48, SD = 0.14, range = 0.03–0.87
CBCL internalizing score^^^	Mean = 4.95, SD = 5.60, range = 0–50	Mean = 4.73, SD = 5.46, range = 0–50
CBCL externalizing score^^^	Mean = 3.91, SD = 5.51, range = 0–50	Mean = 3.49, SD = 5.05, range = 0–44

All measures, including age, are reported using data from ABCD Study follow-up year 2 collection, except for the PDS slope, which was calculated using all available participant data in the five available collections (baseline, follow-up years 1–4).PDS = Pubertal development scale (Petersen et al., 1988); CBCL = Child behavior checklist (Achenbach, 1999).

* PDS scores were missing for *n* = 38 youth in the brain models. All youth in the behavioral models had PDS scores.

^ CBCL scores were missing for *n* = 2,697 in the behavioral models. All youth in the brain models had CBCL scores.

### Measures

#### Resting state fMRI

Resting state fMRI was collected in 5 min long scans where participants passively viewed a fixation cross. Four scans were collected from each participant, yielding up to 20 min of resting state fMRI.

#### Perceived pubertal development

Perceived physical changes associated with pubertal development were assessed using parent-report on the Pubertal Development Scale (PDS, Petersen et al., 1988) to approximate pubertal status (i.e., stage). The PDS asks parents about physical changes in their children’s height, body hair, skin, as well as sex-specific changes for males and females. Pubertal status data from baseline through follow-up year four (up to five annual timepoints of puberty data) was used to assess the rate at which pubertal status changed across time, termed ‘pubertal tempo’ (see Statistical analyses). For the current study, we used pubertal status reported during follow-up year two and pubertal tempo. We chose to use the PDS score rather than a categorical pubertal stage level in order to capture variance dimensionally across individuals.

#### Mental health problems

Mental health symptoms and severity were assessed using parent-report on the Child Behavior Checklist (CBCL, Achenbach, 1999). The CBCL probes a broad range of mental health-related behaviors that fall into eight different subscales. For the current study, we used the CBCL unstandardized summary scores for internalizing and externalizing problems. Internalizing problems are a category of symptoms of mental health problems including worry, fear, rumination, low self-esteem, and sadness (Achenbach, 1999). Externalizing problems are another category of symptoms that includes disruptive behaviors, aggression, and impulsivity. The CBCL internalizing problems score is a sum of items across the following subscales: anxious/depressed, withdrawn/depressed, and somatic complaints. The CBCL externalizing problems score is a sum of items across the rule breaking and aggressive behavior subscales (Achenbach, 1999).

#### Sleep disturbances

Sleep behaviors and problems were assessed with parent-report on the Sleep Disturbance Scale for Children (Bruni et al., 1996). The Sleep Disturbance Scale for Children includes six subscales reflecting different sleep problems: problems initiating or maintaining sleep, sleep breathing problems, arousal problems, sleep–wake transition problems, somnolence problems, and hyperhidrosis. These scales culminate in a ‘total sleep disturbance’ measure that was used for the current study.

#### Demographics

Age, sex, race/ethnicity, parent marital status, combined family income, highest parent education, and neighborhood disadvantage index were also assessed. To align with other recent studies (Rakesh et al., 2021; Sripada et al., 2021), we calculated a composite socioeconomic status score with the following variables: family income, parent education, and neighborhood disadvantage index. As family income was reported categorically with varying bin sizes, we took the log of the average value in each bin, with the exception of the highest value in the lowest bin and the lowest value in the highest bin. Additionally, parent education was converted into a number representing the years of education. Neighborhood disadvantage index was calculated from each participant’s home address (Kind et al., 2014; Fan et al., 2021). In a confirmatory factor analysis in the lavaan R package (Rosseel, 2012) we loaded the three variables on one common latent factor and then extracted the latent factor score for each subject.

### Image acquisition and processing

Neuroimaging data for the current study were downloaded from the ABCD-BIDS collection 3165 from the NIMH Data Archive (NDA). Collection details of the neuroimaging data can be found at (Casey et al., 2018).

Image processing using current standard best practices was carried out using the ABCD-BIDS pipeline version 0.1.3 (Feczko et al., 2021). Steps included registration of the anatomical and functional data, distortion and nonlinearity corrections, and image alignment. Images were further processed in individualized surface spaces using the DCANBOLDProcessing pipeline (Miranda-Domínguez et al., 2020). Pipeline steps included temporal filtering, nuisance regression including motion parameters, interpolation across high motion timepoints, bandpass filtering, and then removal of high motion timepoints (filtered framewise displacement >0.2). Full processing details can be found in (Feczko et al., 2021).

### Functional connectivity and graph metrics

Resting state runs were concatenated for each individual and individuals with less than 5 min of data remaining after removing high motion timepoints were excluded from the analyses. Blood oxygen level dependent (BOLD) timeseries were extracted with a widely used functional atlas included in the ABCD-BIDS dataset, which contains 333 surface brain regions, or ‘nodes,’ comprising 13 functional networks from a previously defined atlas (Gordon et al., 2016). Parcellated timeseries were correlated using Pearson pairwise correlation resulting in a 333×333 correlation matrix representing the whole brain functional connectivity profile. Correlation matrices were Fisher z-transformed creating weighted brain graphs with each transformed correlation value representing a functional connection between two nodes.

We estimated brain network organization using graph metrics that measure integration and segregation at the whole brain and network level. Integration refers to stronger connectivity between disparate networks, while segregation refers to separation of distinct networks that have stronger connectivity within than between (Sporns, 2013).

To measure whole brain organization we used global efficiency and modularity. Global efficiency is a measure of integration across the whole brain based on the shortest path length from one node to another (Latora and Marchiori, 2001; Achard and Bullmore, 2007). Higher values indicate greater whole brain integration. Modularity is a measure of how the whole brain splits into distinct networks or modules that are characterized by stronger within- than between-network connectivity (Newman, 2003). Higher values indicate greater whole brain segregation.

To measure organization of particular networks, we used system segregation. System segregation indexes how segregated a set of networks are from each other and is calculated as the difference between within- and between-network connections divided by within-network connections (Chan et al., 2014). We calculated system segregation between the default mode and dorsal attention networks based on previous work in this sample (Yang et al., 2022); thus higher values indicate greater segregation between these two networks.

We calculated global efficiency and modularity on brain graphs that were thresholded at *z* = 0 leaving only the positive connections. Separately, we calculated system segregation on fully signed and weighted brain graphs including all positive and negative connections.

### Statistical analyses

Pubertal tempo: We used a longitudinal mixed effects model to extract the rate of change in pubertal status across age for each participant. Prior to entry in the longitudinal model, age was scaled, such that each 1-point increase corresponded to one standard deviation (8.02 months). We used data from the baseline collection through the follow-up-year-4 collection resulting in up to five timepoints per participant.

We then ran a series of linear regressions: First, we fit a linear regression to test how total sleep disturbance (dependent variable) was related to pubertal status and pubertal tempo (independent variables; 1 model). To control for multiple comparisons and ensure the family-wise error rate is 0.05, the two *p*-values were corrected with the Benjamini-Hochberg false discovery rate (FDR) at *q* < 0.05 (Benjamini and Hochberg, 1995). All *N* = 10,596 participants were included in this linear regression.

Then, we fit linear regressions to test how CBCL scores (dependent variables) were related to total sleep disturbance with separate moderating effects of pubertal status and pubertal tempo (independent variables). Models for CBCL internalizing problems and CBCL externalizing problems were run separately (two models). The two *p*-values of interest (i.e., one for each interaction) were FDR-corrected at *q* < 0.05 separately for the internalizing and externalizing problem models. All *N* = 10,596 participants were included in these linear regressions.

Finally, we fit linear regressions to test how a three-way interaction between three continuous measures (total sleep disturbance, a pubertal measure, and a brain network metric) related to one continuous outcome measure (a CBCL score). The pubertal measure was either pubertal status or pubertal tempo. The brain network metric was either global efficiency, modularity, or system segregation between the default mode and dorsal attention networks. The CBCL score was either internalizing or externalizing problems. The three-way interactions tested used this model structure:


$$\begin{array}{l} \mathrm{CBCL}\;\mathrm{score}\sim \mathrm{Total}\;\mathrm{sleep}\;{\mathrm{disturbances}}^{*}\mathrm{Pubertal}\;{\mathrm{measure}}^{*}\\ \;\mathrm{Brain}\;\mathrm{network}\;\mathrm{metric} \end{array}$$

Models were run separately for each combination of CBCL score (i.e., internalizing problems, externalizing problems), pubertal measure (i.e., pubertal status, pubertal tempo), and brain network metric (i.e., global efficiency, modularity, system segregation; 12 models). Specifically, these models allowed for us to ask how pubertal development and total sleep disturbance jointly shape relationships between brain network metrics and CBCL scores. The main effects of all interaction terms were also included independently in the model. The p-values of interest were FDR-corrected at *q* < 0.05 separately for each CBCL score and pubertal measure, such that the correction was applied to four sets of three *p*-values each. Participants with brain data, *N* = 2,901 participants, were included in these linear regressions.

We used a progressive model fitting approach to select the covariates. Covariates were tested one at a time in this order: age, sex, collection site, socioeconomic status, race/ethnicity, parent marital status. Each covariate that improved the model fit was retained, while covariates that did not improve model fit were left out and the progressive model fitting procedure continued without that covariate. Any covariate that significantly improved model fit in any model, was retained in all final models so that covariates were identical across all models. Across all models age did not improve model fit and was left out of the final models. All models were run with fixed effect covariates of sex, collection site, socioeconomic status, race/ethnicity, and parent marital status.

## Results

### Puberty related to sleep disturbances

Sleep disturbances were predicted by pubertal status (*b* = 0.81, adjusted-*p* < 0.001), but not pubertal tempo (*b* = −0.86, adjusted-*p* = 0.239; Supplementary Table 1). Youth with more advanced pubertal status (i.e., youth with a PDS score greater than their peers) reported more sleep disturbances. As there was a significant effect of sex, we ran the models separately for males and females and the effects were largely the same as in the whole sample (Supplementary Table 2).

### Puberty moderates the relationships between sleep disturbances and mental health burden

#### Internalizing problems

**Figure 1:**
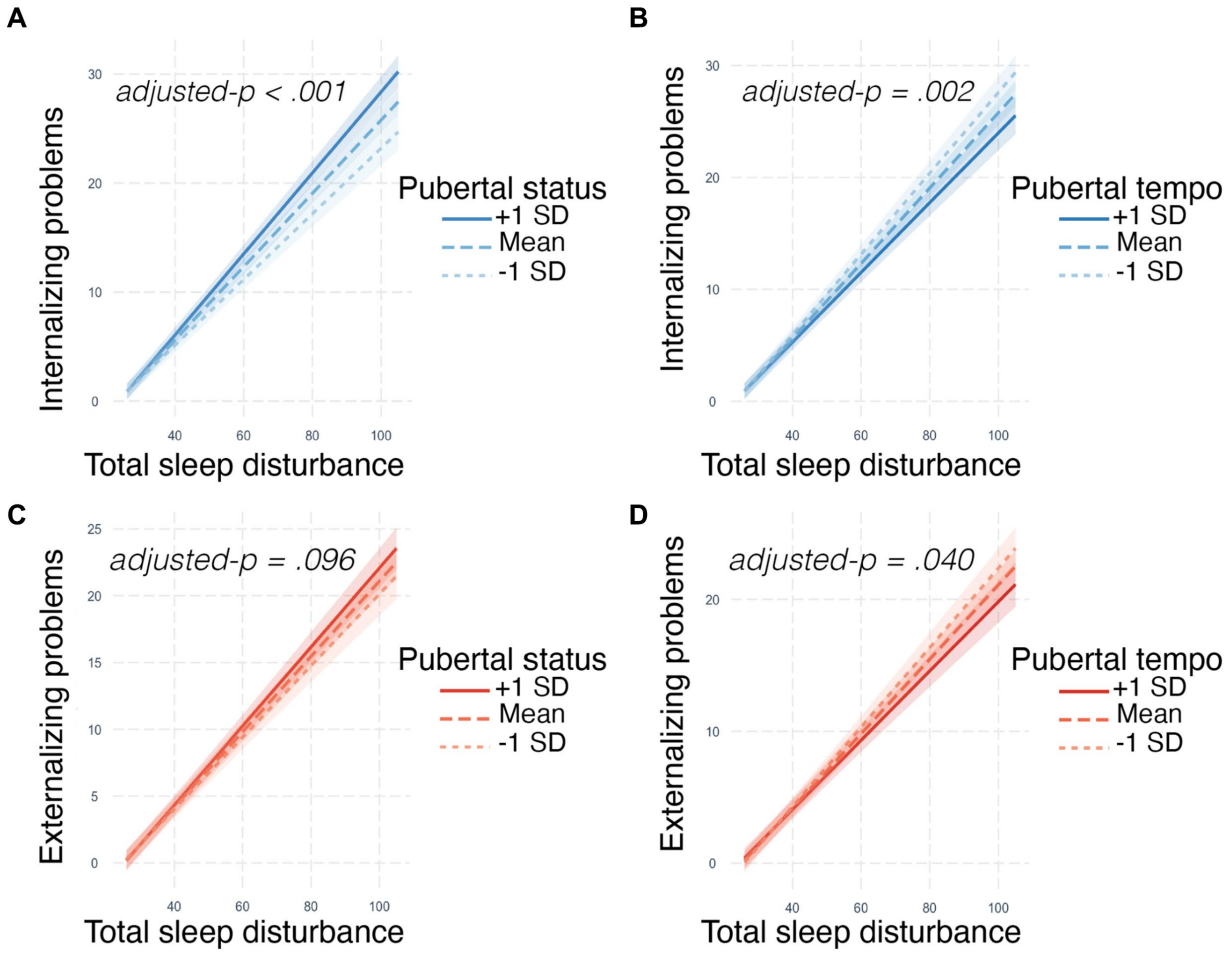
Pubertal development modulates the relationships between sleep disturbances and internalizing and externalizing problems. Pubertal status and pubertal tempo modulate how sleep disturbances relate to **(A,B)** internalizing and **(C,D)** externalizing problems. Stronger relationships between sleep disturbances and internalizing/externalizing problems were observed in individuals with more advanced pubertal status (e.g., +1 SD) and slower pubertal tempo (e.g., −1 SD). Plots were created with the *interactions* R package (Long, 2022). Internalizing and externalizing problems assessed on the CBCL (Achenbach, 1999); Total sleep disturbances assessed on the Sleep Disturbance Scale for Children (Bruni et al., 1996); Pubertal status and tempo derived from the PDS (Petersen et al., 1988); SD = standard deviation. 95% confidence interval ribbons are displayed on each regression line.

The effect of sleep disturbances on internalizing problems differed by pubertal status (*b* = 0.05, adjusted-*p* < 0.001) and pubertal tempo (*b* = −0.18, adjusted-*p* = 0.002). Specifically, individuals with more sleep disturbances exhibited more internalizing problems, and this relationship was exacerbated for individuals with more advanced pubertal status and individuals with slower pubertal tempo (Table 2; Figures 1A,B).

**Table 2:** Interactions between puberty and sleep predicting mental health problems.

Outcome measure	Interaction term 1: sleep measure	Interaction term 2: pubertal measure	*N*	*b*	CI	*p*	Adjusted-*p*
Internalizing problems	Total sleep disturbances	Pubertal status	7,899	0.05	0.03–0.07	**<0.001**	**<0.001**
Internalizing problems	Total sleep disturbances	Pubertal tempo	7,899	−0.18	−0.30–−0.06	**0.002**	**0.002**
Externalizing problems	Total sleep disturbances	Pubertal status	7,899	0.02	−0.00–0.04	0.096	0.096
Externalizing problems	Total sleep disturbances	Pubertal tempo	7,899	−0.14	−0.26–−0.02	**0.020**	**0.040**

Results are reported for the whole sample. N = number of observations; b = unstandardized beta estimates; CI = 95% confidence interval. Bold values denote p-values < 0.05.

#### Externalizing problems

The effect of sleep disturbances on externalizing problems differed by pubertal tempo (*b* = −0.14, adjusted-*p* = 0.040), but not pubertal status (though it was trending; *b* = 0.02, adjusted-*p* = 0.096). Specifically, more sleep disturbances related to more externalizing problems and this relationship was stronger for individuals with slower pubertal tempo (Table 2; Figures 1C,D).

As there were significant effects of sex in both models, we ran all models separately for males and females (Supplementary Table 3).

### Three-way interactions of puberty, sleep, and resting state brain network organization predicting mental health problems

#### Internalizing problems

All three-way interactions with pubertal status predicting internalizing problems were not significant (all adjusted-*p*-values > 0.986). That is, the effect of each tested brain metric (global efficiency, modularity, system segregation of default mode and dorsal attention networks) on internalizing problems did not differ by sleep disturbances and pubertal status.

**Figure 2:**
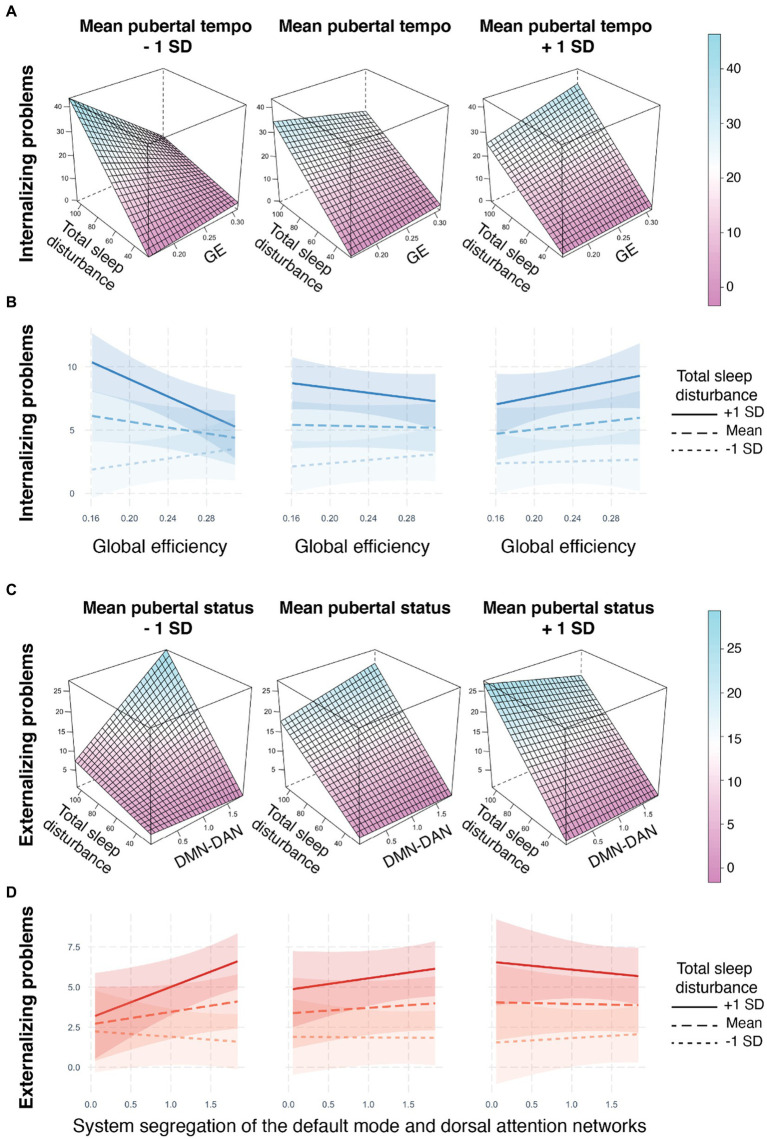
Metrics of resting state brain network organization relate to internalizing and externalizing problems through three-way interactions with sleep disturbances and pubertal development. **(A,B)** The relationship between global efficiency and internalizing problems is modulated by pubertal tempo and sleep disturbances. For individuals with slower pubertal tempo (e.g., −1 SD) and greater sleep disturbances (solid line), less global efficiency was related to higher internalizing problems. **(C,D)** The relationship between system segregation of the default mode and dorsal attention networks and externalizing problems is modulated by pubertal status and sleep disturbances. For individuals with less advanced pubertal status (e.g., −1 SD) and greater sleep disturbances (solid line), more system segregation was related to higher externalizing problems. 3D plots were created with the *lattice* R package (Sarkar, 2008). No covariates were included in the construction of the 3D plots. Interaction line plots were created with the *interactions* R package (Long, 2022). Internalizing and externalizing problems assessed on the CBCL (Achenbach, 1999); Total sleep disturbances assessed on the Sleep Disturbance Scale for Children (Bruni et al., 1996); Pubertal status and tempo derived from the PDS (Petersen et al., 1988); GE = global efficiency, DMN-DAN = system segregation between the default mode and dorsal attention networks, SD = standard deviation.

The effect of global efficiency on internalizing problems differed jointly by sleep disturbances and pubertal tempo (*b* = 14.20, adjusted-*p* = 0.048; Figures 2A,B, Table 3). A post-hoc simple slopes analysis (Supplementary Table 4) revealed a significant relation at the slowest pubertal tempo (e.g., −1 standard deviation from the mean), such that youth with more sleep disturbances exhibited a negative relationship between global efficiency and internalizing problems. At the mean and high (e.g., +1 standard deviation from the mean) pubertal tempos, there were no significant simple slopes and the relationships between brain and internalizing problems were more weakly modulated by sleep (Figures 2A–D). To summarize, this three-way interaction indicates that pubertal tempo moderates the relation between sleep disturbances and whole-brain integration to predict internalizing problems, and this modulation is strongest in individuals with a slower pubertal tempo.

**Table 3:** Three-way interactions predicting mental health problems.

Outcome measure	Interaction term 1: brain metric	Interaction term 2: pubertal measure	Interaction term 3: sleep measure	*N*	*b*	CI	*p*	Adjusted-*p*
Internalizing problems	Modularity	Pubertal status	Total sleep disturbances	2,863	−0.04	−1.18–1.10	0.950	0.986
Internalizing problems	Global efficiency	Pubertal status	Total sleep disturbances	2,863	0.09	−2.14–2.32	0.938	0.986
Internalizing problems	DMN-DAN system segregation	Pubertal status	Total sleep disturbances	2,863	0.00	−0.11–0.11	0.986	0.986
Internalizing problems	Modularity	Pubertal tempo	Total sleep disturbances	2,901	−2.32	−8.19–3.54	0.437	0.656
Internalizing problems	Global efficiency	Pubertal tempo	Total sleep disturbances	2,901	14.20	2.64–25.76	**0.016**	**0.048**
Internalizing problems	DMN-DAN system segregation	Pubertal tempo	Total sleep disturbances	2,901	−0.12	−0.74–0.49	0.694	0.694
Externalizing problems	Modularity	Pubertal status	Total sleep disturbances	2,863	−0.83	−1.95–0.28	0.141	0.211
Externalizing problems	Global efficiency	Pubertal status	Total sleep disturbances	2,863	−0.35	−2.52–1.82	0.753	0.753
Externalizing problems	DMN-DAN system segregation	Pubertal status	Total sleep disturbances	2,863	−0.14	−0.25–−0.04	**0.009**	**0.028**
Externalizing problems	Modularity	Pubertal tempo	Total sleep disturbances	2,901	−0.77	−6.47–4.93	0.792	0.792
Externalizing problems	Global efficiency	Pubertal tempo	Total sleep disturbances	2,901	3.35	−7.90–14.60	0.560	0.792
Externalizing problems	DMN-DAN system segregation	Pubertal tempo	Total sleep disturbances	2,901	−0.49	−1.09–0.10	0.104	0.311

Three-way interaction results are reported for the whole sample. N = number of observations; b = unstandardized beta estimates; CI = 95% confidence interval. Bold values denote p-values < 0.05.

The effect of the other two brain metrics (modularity and system segregation of default mode and dorsal attention networks) on internalizing problems did not differ by sleep disturbances and pubertal tempo (all adjusted-*p*-values > 0.656).

#### Externalizing problems

The effect of system segregation of the default mode and dorsal attention networks on externalizing problems differed jointly by sleep disturbances and pubertal status (*b* = −0.14, adjusted-*p* = 0.028; Figures 2C,D, Table 3). A post-hoc simple slopes analysis (Supplementary Table 5) revealed a significant relation at the least advanced pubertal status (e.g., −1 standard deviation from the mean), such that youth with more sleep disturbances exhibited a more positive relationship between system segregation of the default mode and dorsal attention networks and externalizing problems. At the mean and high (e.g., +1 standard deviation from the mean) pubertal status there were no significant simple slopes and the relationships between brain and externalizing problems were more weakly modulated by sleep (Figures 2C,D). To summarize, this three-way interaction indicates that puberty moderates the relation between sleep and network segregation between the default mode and dorsal attention networks to predict externalizing problems and this modulation is strongest in individuals with a less advanced pubertal status.

The effect of the other two brain metrics (global efficiency, modularity) on externalizing problems did not differ by sleep disturbances and pubertal status (all adjusted-*p*-values > 0.211).

Additionally, the effect of each tested brain metric (global efficiency, modularity, system segregation of default mode and dorsal attention networks) on externalizing problems did not differ by sleep disturbances and pubertal tempo (all adjusted-*p*-values > 0.311).

As there were significant effects of sex in each model, we ran all models separately for males and females (Supplementary Tables 6, 7).

## Discussion

In a large sample of youth at various stages of puberty we found links between perceived pubertal development, sleep disturbances, brain function, and mental health. We found that generally more advanced pubertal status was related to increased sleep disturbances and, additionally, strengthened the relationship between sleep disturbances and internalizing problems. Slower pubertal tempo, or a slower rate of pubertal progression across age, also led to stronger relationships between sleep disturbances and both internalizing and externalizing problems. Of particular interest, we found that the convergence of sleep disturbances and pubertal development shaped the relationship between resting state functional brain network organization and mental health problems. Specifically, for individuals with slow pubertal tempo and more sleep disturbances, whole brain integration (global efficiency) was protective against internalizing problems. Similarly, for individuals with less advanced pubertal status and more sleep disturbances, less segregation between the default mode and dorsal attention networks (system segregation) was protective against externalizing problems.

While each of the factors we examined (sleep disturbances, irregular pubertal development, disrupted brain network organization) has been identified as a risk factor for mental health problems (Dahl, 1999; Ge and Natsuaki, 2009; Di Martino et al., 2014; Mendle, 2014; Goldstone et al., 2020; Mendle et al., 2020; Cui et al., 2022), our findings provide new evidence for the importance of interactions between risk factors. Specifically, we highlight the need for consideration of pubertal development and sleep quality when trying to understand how brain function is linked to internalizing and externalizing problems during adolescence, a developmental period during which there is a rise in mental health problems (Costello et al., 2011; Mendle, 2014). We also acknowledge there are other factors that contribute to this relationship as well, such as media use (Alonzo et al., 2021), which could be further explored to characterize the complex systems linking sleep problems and mental health across adolescence.

### Interactions between networks supporting self-referential processing and attention could be a key mechanism in the link between sleep and mental health problems, particularly for vulnerable youth

Previous work in the ABCD sample (Owens et al., 2020; Lees et al., 2021; Yang et al., 2022; Hehr et al., 2023) found functional connectivity between the default mode and dorsal attention networks was related to shorter sleep, more sleep disturbances, and mental health problems. Additionally, Yang et al. (2022) found that functional connectivity between the default mode and dorsal attention networks mediated the relationship between mental health and sleep disturbances. Building off this work, we applied graph theoretical techniques to examine how the segregation, or separation, of these two networks relates to mental health problems via interactions with sleep disturbances and puberty. We found that for those with less advanced pubertal status and elevated sleep disturbances, decreased segregation of these two networks was a protective factor against elevated externalizing problems. The default mode network, which putatively supports internally-focused attention and self-referential processing, has been suggested to be a key brain network involved in risk for psychopathology (Menon, 2011). Indeed, its connectivity to other networks has been shown to relate to a broad range of mental health problems (Andreescu et al., 2014; Hu et al., 2017; Xia et al., 2018; Scalabrini et al., 2020; Harikumar et al., 2021). Disrupted connectivity between the default mode and dorsal attention networks may underlie the disrupted balance between internally-and externally-directed attention, which is thought to be a primary mechanism of brain dysfunction in attention-deficit/hyperactivity disorder (ADHD; Sonuga-Barke and Castellanos, 2007). Relatedly, youth with ADHD and other externalizing disorders often have worse sleep disturbances (Shanahan et al., 2014; Becker, 2020) and are at risk for developing comorbid internalizing disorders in adolescence (Biederman et al., 2010; Yoshimasu et al., 2012; Becker, 2019). Taken together, disrupted segregation of default mode and attention brain networks seen in attention and externalizing disorders, could be a mechanism by which poor sleep exacerbates mental health problems. Here, we extend this framework to show how pubertal status interacts with sleep disturbances and this neurobiological mechanism—segregation of default mode and dorsal attention networks—to create a particularly vulnerable circumstance for youth.

### Pubertal status and tempo offer complimentary clues as to when sleep-related interventions might be most needed

Interventions on sleep behaviors in adolescents are clearly needed (Blake et al., 2019). Behavioral interventions, such as sleep education, sleep extension, and cognitive behavioral therapy, have been tested with so far mixed results (Paavonen et al., 2016; Blake et al., 2017; Åslund et al., 2018). Legislative shifts to change school start times have also been introduced to improve sleep for adolescents (Lee et al., 2017). Fundamental questions in understanding interventions and supports include - when and for who are these interventions most important? We examined both pubertal status and pubertal tempo as different sources of timing information to clarify when interactions between puberty, sleep disturbances, brain function, and mental health are the strongest. We found evidence that both pubertal status and pubertal tempo are important to consider. Our findings suggest that when focusing on late middle childhood through early adolescence, having a less advanced pubertal stage and going through puberty slower could both be increased risk factors for entanglement of sleep disturbances and mental health problems via brain organization. Those individuals might benefit the most from targeted sleep interventions or from delayed school-start times. One important caveat to consider is that sleep disturbances are linked to mental health problems throughout adolescence (Dahl, 1999; Owens et al., 2014) and targeted sleep interventions would likely benefit many youth. Our work only covers a snapshot of early adolescence, where we do find that within that timeframe there are specific risk factors that make some youth especially vulnerable to sleep disturbances, and likely have the biggest need for intervention. We look forward to more studies which will examine these relationships across adolescence to help confirm particular windows of timing and candidates who would benefit most from intervention.

## Conclusion

Both pubertal status and tempo interact with sleep disturbances and functional brain network organization to predict mental health problems. Connectivity of large-scale brain networks, both at the whole-brain level and particular networks involving self-referential processing and attention, are neurobiological phenomena that interact with sleep disturbances and puberty to predict mental health problems. This work highlights the importance of considering pubertal development and common developmentally-related challenges, such as sleep disturbances, when investigating the link between brain function and mental health problems. This work also suggests that both pubertal status and tempo are important to consider when designing interventions for sleep disturbances in youth.

## Data availability statement

Publicly available datasets were analyzed in this study. This data can be found at: ABCD Study Data Release 5.0 doi: 10.15154/8873-ZJ65.

## Ethics statement

The studies involving humans were approved by University of California, San Diego, and local site institutional review boards. The studies were conducted in accordance with the local legislation and institutional requirements. Written informed consent for participation in this study was provided by the participants’ legal guardians/next of kin.

## Author contributions

MM: Conceptualization, Writing – original draft, Writing – review & editing, Investigation, Methodology. TN: Conceptualization, Writing – original draft, Writing – review & editing.

## Footnotes

^1^https://collection3165.readthedocs.io/en/stable/
