# Supplementary material for: Puberty interacts with sleep and brain network organization to predict mental health
Source: Front Hum Neurosci. 2024 Sep 27;18:1379945. doi: 10.3389/fnhum.2024.1379945 (PMC11466844; doi:10.3389/fnhum.2024.1379945)
Supplement: Supplementary file 1 [file Table_1.docx]

**Supplementary materials** for ‘*Puberty interacts with sleep and brain network organization to predict mental health’*

*Mackenzie E. Mitchell & Tehila Nugiel*

**Results**

1. Puberty related to sleep disturbances

**Supplementary Table 1.** Pubertal measures predicting total sleep disturbance in the whole sample.

| *Sample* | *Outcome* | *Predictors* | *N* | *b* | *CI* | *p* | *adjusted-p* |
| --- | --- | --- | --- | --- | --- | --- | --- |
| Whole sample | Total sleep disturbance | Pubertal status | 10596 | 0.81 | 0.52 – 1.11 | **<.001** | **<.001** |
| Whole sample | Total sleep disturbance | Pubertal tempo | 10596 | -0.86 | -2.28 – 0.57 | .239 | .239 |
| Whole sample | Total sleep disturbance | Sex [F] | 10596 | -0.66 | -1.02 – -0.30 | **<.001** | - |

Results are reported for the whole sample. *N* = number of observations; *b* = unstandardized beta estimates; CI = 95% confidence interval

**Supplementary Table 2.** Pubertal measures predicting total sleep disturbance in the female and male samples.

| *Sample* | *Outcome* | *Predictor* | *N* | *Estimates* | *CI* | *p* |
| --- | --- | --- | --- | --- | --- | --- |
| Females only | Total sleep disturbance | Pubertal status | 5023 | 1.01 | 0.64 – 1.38 | **<.001** |
| Females only | Total sleep disturbance | Pubertal tempo | 5023 | -1.11 | -3.13 – 0.91 | .280 |
| Males only | Total sleep disturbance | Pubertal status | 5573 | 0.60 | 0.12 – 1.07 | **.014** |
| Males only | Total sleep disturbance | Pubertal tempo | 5573 | -0.67 | -2.75 – 1.40 | .525 |

Results are reported for the whole sample. *N* = number of observations; *b* = unstandardized beta estimates; CI = 95% confidence interval

2. Puberty moderates the relationships between sleep disturbances and mental health burden

**Supplementary Table 3.** Interactions between puberty and sleep predicting mental health problems in the female and male samples.

| *Sample* | *Outcome measure* | *Interaction term 1: Sleep measure* | *Interaction term 2: Pubertal measure* | *N* | *b* | *CI* | *p* |
| --- | --- | --- | --- | --- | --- | --- | --- |
| Females only | Internalizing problems | Total sleep disturbance | Pubertal status | 3762 | 0.06 | 0.02 – 0.09 | **.001** |
| Females only | Internalizing problems | Total sleep disturbance | Pubertal tempo | 3762 | -0.22 | -0.41 – -0.04 | **.020** |
| Females only | Externalizing problems | Total sleep disturbance | Pubertal status | 3762 | 0.05 | 0.02 – 0.08 | **.003** |
| Females only | Externalizing problems | Total sleep disturbance | Pubertal tempo | 3762 | -0.06 | -0.24 – 0.12 | .507 |
| Males only | Internalizing problems | Total sleep disturbance | Pubertal status | 4137 | 0.02 | -0.02 – 0.05 | .317 |
| Males only | Internalizing problems | Total sleep disturbance | Pubertal tempo | 4137 | -0.18 | -0.34 – -0.02 | **.029** |
| Males only | Externalizing problems | Total sleep disturbance | Pubertal status | 4137 | 0.03 | -0.01 – 0.07 | .209 |
| Males only | Externalizing problems | Total sleep disturbance | Pubertal tempo | 4137 | -0.10 | -0.28 – 0.07 | .248 |

Results are reported for the whole sample. *N* = number of observations; *b* = unstandardized beta estimates; CI = 95% confidence interval

3. Three-way interactions of puberty, sleep, and resting state brain network organization predicting mental health problems

The interactions package in R (Long, 2022) was used to conduct a simple slopes analysis for the models with significant three-way interactions. First, internalizing problems were predicted by a three-way interaction between pubertal tempo, sleep disturbance, and global efficiency (**Supplementary Table 4**).

Second, externalizing problems were predicted by a three-way interaction between pubertal status, sleep disturbance, and system segregation of the DMN/DAN (**Supplementary Table 5**).

**Supplementary Table 4.** Simple slopes for the three-way interaction between pubertal tempo, sleep disturbance, and global efficiency predicting internalizing symptoms in the whole sample.

| *Pubertal tempo value* | *Total sleep disturbance value* | *b* | *SE* | *t* | *p* |
| --- | --- | --- | --- | --- | --- |
| 0.35 (- 1 SD) | 28.37 (-1 SD) | 10.95 | 11.34 | 0.97 | .33 |
|  | 35.96 (Mean) | -11.60 | 8.31 | -1.39 | .16 |
|  | 43.55 (+1 SD) | -34.14 | 11.73 | -2.91 | **.00** |
| 0.48 (Mean) | 28.37 (-1 SD) | 6.46 | 8.28 | 0.78 | .44 |
|  | 35.96 (Mean) | -1.54 | 6.20 | -0.25 | .80 |
|  | 43.55 (+1 SD) | -9.54 | 8.71 | -1.10 | .27 |
| 0.62 (+ 1 SD) | 28.37 (-1 SD) | 1.97 | 11.74 | 0.17 | .87 |
|  | 35.96 (Mean) | 8.52 | 8.36 | 1.02 | .31 |
|  | 43.55 (+1 SD) | 15.07 | 12.44 | 1.21 | .23 |

*b* = unstandardized beta estimate; SE = standard error

**Supplementary Table 5.** Simple slopes for the three-way interaction between pubertal status, sleep disturbance, and system segregation of the DMN/DAN predicting externalizing symptoms in the whole sample.

| *Pubertal status value* | *Total sleep disturbance value* | *b* | *SE* | *t* | *p* |
| --- | --- | --- | --- | --- | --- |
| 1.42 (- 1 SD) | 28.41 (-1 SD) | -0.36 | 0.72 | -0.50 | .62 |
|  | 35.94 (Mean) | 0.78 | 0.62 | 1.26 | .21 |
|  | 43.47 (+1 SD) | 1.91 | 0.78 | 2.45 | **.01** |
| 2.13 (Mean) | 28.41 (-1 SD) | -0.03 | 0.62 | -0.05 | .96 |
|  | 35.94 (Mean) | 0.34 | 0.55 | 0.62 | .54 |
|  | 43.47 (+1 SD) | 0.71 | 0.64 | 1.11 | .27 |
| 2.83 (+ 1 SD) | 28.41 (-1 SD) | 0.29 | 0.72 | 0.40 | .69 |
|  | 35.94 (Mean) | -0.10 | 0.62 | -0.16 | .88 |
|  | 43.47 (+1 SD) | -0.48 | 0.78 | -0.62 | .53 |

*b* = unstandardized beta estimate; SE = standard error

**Supplementary Table 6.** Three-way interactions between puberty, sleep, and brain metrics predicting internalizing symptoms in the female and male samples.

| *Sample* | *Outcome measure* | *Interaction term 1: Brain metric* | *Interaction term 2: Pubertal measure* | *Interaction term 1: Sleep measure* |  | *N* | *b* | *CI* | *p* |
| --- | --- | --- | --- | --- | --- | --- | --- | --- | --- |
| Females only | Internalizing problems | Modularity | Pubertal status | Total sleep disturbance |  | 1438 | 0.14 | -1.61 – 1.90 | .872 |
| Females only | Internalizing problems | Global efficiency | Pubertal status | Total sleep disturbance |  | 1438 | -0.81 | -3.92 – 2.31 | .611 |
| Females only | Internalizing problems | DMN-DAN system segregation | Pubertal status | Total sleep disturbance |  | 1438 | -0.12 | -0.30 – 0.06 | .180 |
| Females only | Internalizing problems | Modularity | Pubertal tempo | Total sleep disturbance |  | 1438 | 7.46 | -2.91 – 17.84 | .158 |
| Females only | Internalizing problems | Global efficiency | Pubertal tempo | Total sleep disturbance |  | 1438 | 31.44 | 13.15 – 49.73 | **.001** |
| Females only | Internalizing problems | DMN-DAN system segregation | Pubertal tempo | Total sleep disturbance |  | 1438 | 0.77 | -0.49 – 2.03 | .229 |
| Males only | Internalizing problems | Modularity | Pubertal status | Total sleep disturbance |  | 1441 | -0.24 | -2.08 – 1.61 | .801 |
| Males only | Internalizing problems | Global efficiency | Pubertal status | Total sleep disturbance |  | 1441 | 1.00 | -2.62 – 4.61 | .589 |
| Males only | Internalizing problems | DMN-DAN system segregation | Pubertal status | Total sleep disturbance |  | 1441 | 0.11 | -0.08 – 0.30 | .245 |
| Males only | Internalizing problems | Modularity | Pubertal tempo | Total sleep disturbance |  | 1441 | -10.94 | -19.87 – -2.01 | **.016** |
| Males only | Internalizing problems | Global efficiency | Pubertal tempo | Total sleep disturbance |  | 1441 | -0.04 | -17.55 – 17.47 | .997 |
| Males only | Internalizing problems | DMN-DAN system segregation | Pubertal tempo | Total sleep disturbance |  | 1441 | -0.65 | -1.57 – 0.27 | .164 |

Results are reported for the whole sample. *N* = number of observations; *b* = unstandardized beta estimates; CI = 95% confidence interval

**Supplementary Table 7.** Three-way interactions between puberty, sleep, and brain metrics predicting externalizing symptoms in the female and male samples.

| *Sample* | *Outcome measure* | *Interaction term 1: Brain metric* | *Interaction term 2: Pubertal measure* | *Interaction term 1: Sleep measure* |  | *N* | *b* | *CI* | *p* |
| --- | --- | --- | --- | --- | --- | --- | --- | --- | --- |
| Females only | Externalizing problems | Modularity | Pubertal status | Total sleep disturbance |  | 1438 | -0.78 | -2.29 – 0.73 | .311 |
| Females only | Externalizing problems | Global efficiency | Pubertal status | Total sleep disturbance |  | 1438 | -1.68 | -4.36 – 1.00 | .219 |
| Females only | Externalizing problems | DMN-DAN system segregation | Pubertal status | Total sleep disturbance |  | 1438 | -0.26 | -0.42 – -0.11 | **.001** |
| Females only | Externalizing problems | Modularity | Pubertal tempo | Total sleep disturbance |  | 1438 | 13.08 | 4.22 – 21.93 | **.004** |
| Females only | Externalizing problems | Global efficiency | Pubertal tempo | Total sleep disturbance |  | 1438 | 4.13 | -11.60 – 19.86 | .607 |
| Females only | Externalizing problems | DMN-DAN system segregation | Pubertal tempo | Total sleep disturbance |  | 1438 | 0.57 | -0.51 – 1.65 | .299 |
| Males only | Externalizing problems | Modularity | Pubertal status | Total sleep disturbance |  | 1441 | -0.56 | -2.58 – 1.46 | .588 |
| Males only | Externalizing problems | Global efficiency | Pubertal status | Total sleep disturbance |  | 1441 | 0.48 | -3.49 – 4.45 | .812 |
| Males only | Externalizing problems | DMN-DAN system segregation | Pubertal status | Total sleep disturbance |  | 1441 | 0.07 | -0.14 – 0.28 | .528 |
| Males only | Externalizing problems | Modularity | Pubertal tempo | Total sleep disturbance |  | 1441 | -9.66 | -19.51 – 0.19 | .055 |
| Males only | Externalizing problems | Global efficiency | Pubertal tempo | Total sleep disturbance |  | 1441 | 2.11 | -17.17 – 21.40 | .830 |
| Males only | Externalizing problems | DMN-DAN system segregation | Pubertal tempo | Total sleep disturbance |  | 1441 | -0.65 | -1.66 – 0.37 | .213 |

Results are reported for the whole sample. *N* = number of observations; *b* = unstandardized beta estimates; CI = 95% confidence interval

**References**

Long, J. A. (2022). interactions: Comprehensive, User-Friendly Toolkit for Probing Interactions. https://cran.r-project.org/package=interactions
